# Implementing primary care behavioral health in Swedish primary care – study protocol for a pragmatic stepped wedge cluster trial

**DOI:** 10.1186/s12875-024-02515-0

**Published:** 2024-08-20

**Authors:** Hanna Israelsson Larsen, Kristin Thomas, Lise Bergman Nordgren, Erica Skagius Ruiz, Kocher Koshnaw, Per Nilsen

**Affiliations:** 1 Department of Health, Medicine and Caring Sciences, Division of Prevention, Rehabilitation and Community Medicine, Linköping University, Linköping, Sweden; 2 Primary Health Care Centre in Cityhälsan Centrum and Department of Health, Medicine and Caring Sciences, Linköping University, Linköping, Sweden; 3 Department of Health, Medicine and Caring Sciences, Division of Society and Health, Linköping University, Linköping, Sweden; 4 Department of Medicine, Faculty of Medicine and Health, Örebro University, Örebro, Sweden; 5 Division of Pychiatry, Region Örebro, Örebro, Sweden; 6 Primary Health Care Centre in Lambohov and Department of Health, Medicine and Caring Sciences, Linköping University, Linköping, Sweden; 7 School of Health and Welfare, Halmstad University, Halmstad, Sweden

**Keywords:** Primary Care, Primary care behavioral health, Implementation, Pragmatic clinical trial, Mental health

## Abstract

**Background:**

Mental health problems represent a large and growing public health concern. Primary care handles most of the patients with mental health problems, but there are many barriers to detection and treatment in this setting, causing under-recognition and under-treatment of patients. The service delivery model Primary Care Behavioral Health (PCBH) shows promise to manage mental health problems in primary care, but more research is needed regarding its effects on multiple levels.

**Methods:**

This project investigates the effectiveness and implementation of a large-scale implementation of PCBH in Region Östergötland, Sweden. The aim is to generate new knowledge concerning the impact of a real-world implementation and use of PCBH in routine primary care. A Pragmatic Stepped-Wedge Cluster Trial will be used: 24 PCBH primary care centres in one region will be compared with 48 standard care centres in three other regions. The model will be implemented sequentially at the PCBH centres according to a staggered timetable. Results will be investigated at patient, staff and organization levels and various forms of data will be collected: (1) local and national registry data; (2) questionnaire data; (3) interview data; and (4) document data.

**Discussion:**

This project investigates the effectiveness and implementation of PCBH in routine primary care. The project could result in improved mental health care for the included patients and contribute to the general good for a wider population who have mental health problems. The project’s study design will make it possible to assess many important effects of the PCBH service delivery model at different levels, providing evidence of the effectiveness (or not) of the PCBH model under routine conditions in primary care. The project has the potential to generate clinically meaningful results that can provide a basis for decisions concerning further implementation and use of the model and thus for future development of mental health care provision in primary care.

**Trial registration:**

NCT05633940, date of registration: 2021–04-21.

## Contributions to the literature

- The project investigates the implementation of a comprehensive service delivery model, comparing 24 primary care centers implementing the model with 48 “standard care” centers, thereby contributing knowledge about real-world implementation.
- A multifaceted evaluation approach is employed to enable an investigation of three levels of the service delivery model, patient, staff and organization levels, thus adding knowledge for implementation science which typically focuses on the staff level.
- A rigorous Stepped-Wedge design is applied, which is not a common design in implementation science, but is appropriate when practical, logistical and resource constraints make it difficult to deliver an intervention en bloc.

## Background

### Introduction

Both mental health problems (e.g., sleep disturbances and stress) and psychiatric disorders (e.g., depression and anxiety disorders) are highly prevalent in today’s society [1–5]. There is a strong connection between detrimental behavioral patterns and decreased mental health. Approximately 25–50% of all patients in primary care worldwide have symptoms attributable to behavioral or mental health problems, with an increasing proportion of symptoms occurring over time [3, 5–7]. These problems restrict individuals’ ability to function, engage in daily activities and maintain social relationships, causing significant suffering for individuals and their families [1, 2]. There is also a well-established link between mental health problems and low quality of life as well as cardiovascular disease, e.g., myocardial infarction and stroke [8, 9].

In Sweden, primary care handles most patients with mental health problems, with 50% to 70% of patients diagnosed with psychiatric diseases being managed in this setting [2, 10]. The corresponding international figure is approximately 60% [7]. Despite the high prevalence of mental health problems, there are many barriers to achieving optimal detection and treatment in primary care [11]. Thus, mental and behavioral health problems represent a major challenge for already strained primary care, where the need for mental health care exceeds the available resources [1–5].

The majority of mental health problems present in primary care can be managed by psychological interventions or even self-care [10]. The recommended evidence-based treatment for mild to moderate mental health problems often is manualized cognitive behavioral therapy (CBT), which focuses on changing both patients’ thoughts and behavioral patterns [12]. However, very few primary care patients, both worldwide and in Sweden, receive CBT, primarily due to a lack of resources [1–3, 6, 13]. There is a shortage of psychotherapists, psychologists and nurses [14], which restricts care opportunities, often resulting in long waiting lists, delays and multiple, diverse contacts before appropriate care is obtained, which may lead to worsening symptoms that ultimately manifest as psychiatric disorders [10, 15, 16]. Another consequence is overmedication with psychotropic drugs [13].

### The primary care behavioral health model

The primary care behavioral health (PCBH) model has attracted considerable interest from primary care organizations both in Sweden and internationally as a promising model for addressing the challenges of behavioral and mental health problems in the primary care population [17–19]. The PCBH model is a team-based primary care approach for managing mental health problems and biopsychosocially influenced health conditions. The model’s main goal is to enhance the primary care team’s ability to manage and treat such problems/conditions, with resulting improvements in primary care services for the entire clinic population. An important premise is to address these problems early on, focusing on helping patients learn skills to improve their functioning before symptoms develop into disease and, for example,reduce their work capacity. To this end, patients must be offered proactive, timely and adequate treatment in primary care [10].

The PCBH model strives to reduce behavioral health problems, thereby promoting overall health in the general population [18]. This objective is aligned with the overarching goals of primary care in Sweden [20], making the model clinically attractive. PCBH can be described as a patient-centered service delivery model based on a multiprofessional team-based approach [17–19]. The PCBH service delivery model has two primary key features that distinguish it from how primary care providers traditionally handle mental health issues, as described below:

- Behavioral health consultants: PCBH emphasizes high accessibility to an on-site behavioral health consultant (BHC) as a routine part of the primary care clinic. This on-site BHC works closely with physicians and assists all staff members at the clinic in addressing mental and behavioral health issues. The BHC works as a generalist and an educator in behavioral health at the primary care center, working in a productive, accessible manner [17–19]. In Sweden, the role of BHC is typically as a psychologist or psychotherapist. The ambition is that patients with mental health problems, if needed, may receive an appointment with a BHC either directly or within a few days. Such appointments can be arranged at the patient’s first visit to the center or immediately after a visit to a physician.
- Brief interventions: Short, timely interventions are offered for mental health problems as soon as these problems affect a patient’s functioning rather than interventions solely based on diagnostic criteria for psychiatric diseases [18]. In clinical practice, these interventions are targeted evidence-based interventions and can be delivered via several different modalities, such as face-to-face on-site meetings or via the internet [21].

### Knowledge gaps

Despite its potential, many aspects of the PCBH model have not been investigated. There have been calls for robust, clinically relevant research on the model, focusing on clinical, financial and operational factors and addressing patient, staff and organization levels [17, 19]. Knowledge is needed concerning the effects of PCBH on clinically relevant patient outcomes in diverse primary care patient populations. It is also important to conduct research to investigate the feasibility of PCBH in smaller or middle-sized primary care centers where resources might be limited. Furthermore, the effects of the PCBH on staff and organization levels, e.g., the model’s impact on waiting lists and the number of patients who receive treatment for mental health problems, are also important to evaluate. Knowledge is also needed about the barriers and facilitators to implementing PCBH in primary care and the acceptability, appropriateness and feasibility of using the model as part of routine work. The costs of implementing and working according to the model and the cost-effectiveness of PCBH are other relevant areas to investigate. The research project described in this protocol paper aims to address the majority of these issues.

### Objectives

The project aim for this Pragmatic Stepped-Wedge Cluster Trial is to generate new knowledge concerning the impact of a real-world implementation of PCBH in routine primary care in Sweden. Twenty-four PCBH primary care centers will be compared with 48 standard care centers. The results will be investigated at the patient, staff and organization levels. Eight research questions (RQs) are addressed (for details, see Table 1):

**Table 1:** Data sources and focus for each research question

Focus	Research question	Data source(s)
Patients	(RQ1) What are the effects of PCBH on patient outcomes compared to standard care?	• National registry data, INERA certification service (Swedish Social Insurance Agency): Patient sick leave due to mental health problems (i.e., sick leave due to ICD10: F00-F99, Z56, Z73F) • Local registry data: Referrals to secondary psychiatric care based on electronic medical records
	(RQ2) What characterizes patients who receive care according to the PCBH model compared to standard care?	Local registry data except where noted • Patient demography (gender, age) • Patient diagnoses F00-F99, Z56, Z73F according to ICD10, including specific diagnoses and combinations of diagnoses • Comorbidity calculated using the Charlson Comorbidity Index • National registry data, Swedish Prescribed Drug Registry national data (National Board of Health and Welfare): Medication with psychotropic drugs coded as N05A, N05B, N05C and N06A according to the ATC system
	(RQ3) How do patients experience the care provided at centers that implement PCBH?	• Interviews: Patient experiences with the behavioral consultant care they have received will be explored with interviews
Staff	(RQ4) How do health care staff members experience working with PCBH in terms of barriers to and facilitators for implementation?	• Interviews: Staff experiences of working with PCBH in terms of e.g., barriers and facilitators for implementation of PCBH in routine care
	(RQ5) How do mental health therapists’ experience their new PCBH-specific role as behavioral health consultants?	• Interviews: Mental health therapists’ experiences of taking on the new role as behavioral health consultants
	(RQ6) How does implementation of the PCBH model affect the work environment?	Questionnaires • Sociodemographic factors: age, gender, profession, year in that profession, time employed at the primary health care center • Staff psychosocial factors: the COPSOQ III questionnaire • Staff work commitment: Utrecht Work Engagement Scale • Exhaustion among co workers: KEDS
Organization	(RQ7) How does PCBH influence accessibility to mental health care in primary care?	All based on local registry data: • Organization and context characteristics: number of employees, number of behavioral health consultants and physicians, number of listed patients, characteristics of the catchment area, care need index (CNI) and adjusted clinical groups • Number of visits to behavioral health consultants • Waiting times to first visit and revisits to behavioral health consultants and physicians • Types of mental health interventions (e.g. focused acceptance and commitment therapy, motivational interviewing, concise CBT, behavioral activation) • Format of delivery (e.g. internet-based or self-help materials) • Number of visits to physicians for patients diagnosed with F00-F99, Z56, Z73F, or prescribed medication with N05A, N05B, N05C and N06A
Economy	(RQ8) What is the cost-effectiveness of implementing and working based on PCBH?	The cost analysis will be based on several data sources, including registry data also used to address RQ1 and written documentation concerning the implementation activities undertaken during the implementation period for each participating primary care center

*Abbreviations: PCBH* Primary care behavioral health, *RQ* Research Question

Patient level:

- (RQ1) What are the effects of PCBH on patient outcomes compared to standard care?
- (RQ2) What characterizes patients who receive care according to the PCBH model compared to standard care?
- (RQ3) How do patients experience the care provided at centers that implement PCBH?

Staff level:

- (RQ4) How do health care staff members experience working with PCBH in terms of barriers to and facilitators of implementation?
- (RQ5) How do mental health therapists' experience their new PCBH-specific role as behavioral health consultants?
- (RQ6) How does implementation of the PCBH model affect the work environment?

Organization level:

- (RQ7) How does PCBH influence accessibility to mental health care in primary care?
- (RQ8) What is the cost-effectiveness of implementing and working based on PCBH?

## Methods

### Study setting

This research project will study the real-world implementation of the PCBH in routine primary care in the Region Östergötland (RÖ), Sweden. The trial will include 24 intervention primary care centers (PCBH) and 48 control centers located in adjacent regions. Implementation sites will be matched with control sites based on the number of listed patients, number of employees (psychologist/psychotherapist and physicians), characteristics of the catchment area, care need index (CNI) and adjusted clinical groups (ACG). All participating sites are situated in southern Sweden in both urban and rural locations.

### Study design

The study is a Pragmatic Stepped-Wedge Cluster Trial, which enables us to study multiple effects on several different levels, encompassing both quantitative and qualitative methods. This design is increasingly used in the evaluation of service delivery models [22]. Pragmatic clinical trials evaluate interventions and service delivery models under real-life routine practice conditions [23]. In cluster trials, preexisting groups, in this case, primary care centers, are allocated to intervention or control groups [24]. In this stepped-wedge design, the model is implemented sequentially at the included centers according to a staggered timetable. Stepped-wedge trials are considered more powerful than traditional parallel studies when clusters are relatively heterogeneous and/or large [25], which is the case in this project. The control centers will be offered the model after the study period. We used the SPIRIT checklist when writing our report.

### Implementation of the model

There is a regional decision that PCBH should be implemented in the whole primary care in RÖ in the south-east region of Sweden, and the research group is working closely with the assigned regional implementation team, which consists of psychologists with special training in PCBH.

**Fig. 1:**
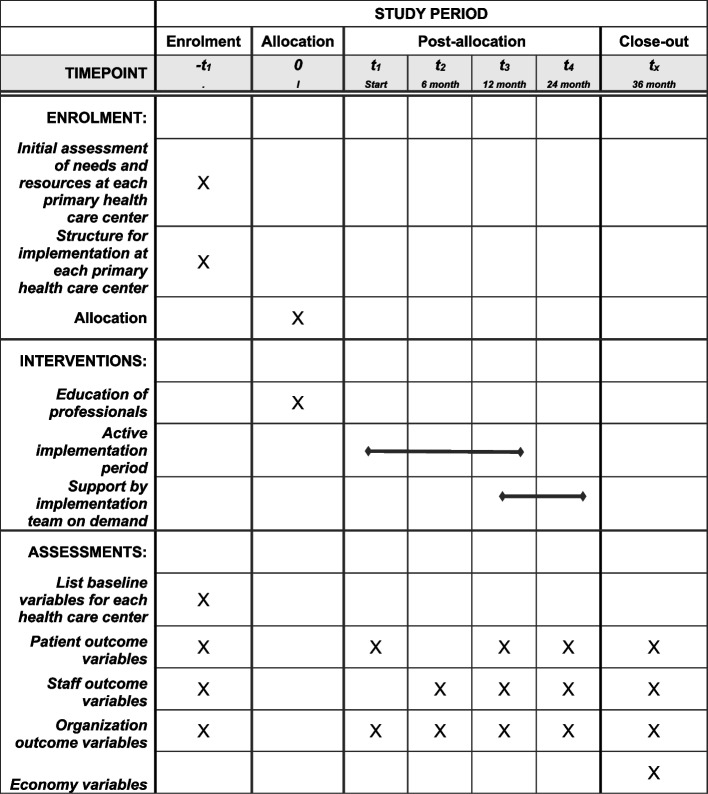
SPIRIT flow diagram. Legends: SPIRIT flow diagram for enrolment, interventions, and assessments. For details regarding assessment and outcome variables, see Table 1

The implementation process will be carried out according to the Swedish Public Health Agency’s “Checklist for implementation with quality” [26]. 1) Initial assessment of needs and resources; 2) structure for implementation; 3) actual implementation; and 4) learn and improve. For each primary health care center, an initial assessment of needs and resources take place, followed by an individually tailored structure for implementation. When implementation starts, all medical staff receive a PCBH education for 1–4 days depending on the profession (e.g., 4 days: staff categories who will take on the role of on-site behavioral health consultants, typically psychologists and psychotherapists; 2 days: nurses; 1 day: physicians). Thereafter, tutoring sessions per profession are being carried out approximately once/month, more often if needed, to promote retention of the PCBH model. The active implementation period for each intervention center is approximately 12 months, during which scheduled lectures and workshops, continuous on-demand support, materials and instructions are provided by the implementation team. Thereafter, further support for maintenance will be offered by the implementation group for at least 24 months according to the needs of each center. All the implementation activities are endorsed and funded by RÖ. See also Fig. 1: SPIRIT flow diagram.

### Logic model

**Fig. 2:**
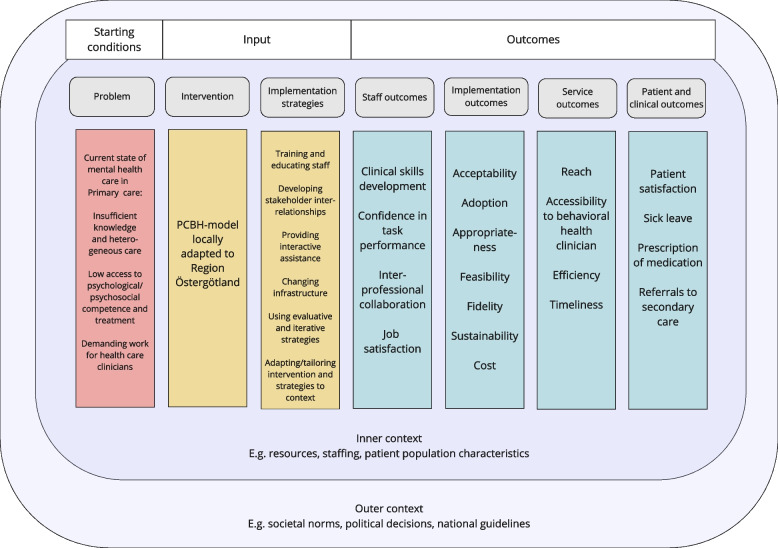
Logic model. Legends: Logic model for the research project, which depicts the relationships between the PCBH activities, the strategies used to support the implementation of the model and the various intended outcomes. Abbreviations: PCBH: primary care behavioral health

A logic model was developed by the research team to describe the project and enable reflection on the assumptions underlying the implementation of the PCBH service delivery model in RÖ; see Fig. 2. The logic model provides information about the problems addressed by the PCBH model and graphically depicts the relationships between the PCBH activities, the strategies used to support the implementation of the model and the various intended outcomes. A logic model is a systematic and visual way to present and share hypothesized descriptions of the chain of causes and effects leading to outcomes of interest of an intervention [27].

First, the logic model identifies the three key problems of the poor current state of behavioral health in primary care globally: problems that the PCBH model is intended to solve or reduce; 1) insufficient knowledge concerning behavioral health care among the primary care staff; 2) poor patient access to relevant behavioral health care; and 3) high work demands for primary care staff that make it difficult to handle behavioral health issues in busy clinical reality [1–3, 6, 13, 28, 29]. These problems are described as the “starting conditions” of the PCBH model.

The “input” in the logic model is provided by the intervention, i.e., the PCBH service delivery model, and the implementation strategies that will be used to facilitate its implementation in routine primary care. The PCBH model and detailed implementation strategies are described elsewhere in the study protocol.

The following four types of “outcomes” are delineated in the logic model: staff, implementation, service and patient outcomes. Implementation, service and patient outcomes are informed by an implementation outcome taxonomy developed by Proctor et al. [30], while staff outcomes are based on insights about the PCBH model, as conveyed in the literature on the model [17–19].

Staff outcomes are intended effects pertaining to the staff in the primary care centers that implement the PCBH model, e.g., improvements with regard to staff members’ self-efficacy in working with behavioral health issues, work environment and better interprofessional collaboration. Implementation outcomes relate to various aspects of the implementation of the PCBH model, e.g., cost and fidelity. Service outcomes are related to desirable service-level effects if the PCBH services are delivered to the expected standard. These effects include the model’s intended improvements in, e.g., accessibility to care and timeliness of receiving care compared to standard care. Patient outcomes are the desired patient effects of receiving care according to the PCBH model. Compared to standard care, the PCBH model is intended to achieve increased patient satisfaction, reduced sick leave, less prescription of medication and fewer referrals to secondary care.

### Participants

#### Primary care center recruitment

Four pilot primary care centers have already been enrolled in the project, to test the protocol. After this pilot enrollment and adjustments of the implementation procedure, the protocol was adjusted to the final version. Following the pilot implementation of PCBH, a total of 24 primary care centers in RÖ will be enrolled in the research project (representing a total of 70% of all centers in this region). According to a staggered timetable, eight centers will be included in Year 1, eight in Year 2 and eight in Year 3, for a total of 24 centers over a three-year period. These 24 primary care centers in RÖ will be matched with 48 control centers from adjacent county councils in Jönköping, Kalmar and Örebro. Matching will be based on the number of listed patients, number of employees, number of behavioral health consultants and physicians and characteristics of the catchment area (CNI and ACG). A similar cluster-based approach has previously been used in research conducted at Linköping University [31].

#### Patient recruitment

The eligible study participants are all adult patients (≥ 18 years) who have either received any of the following International classification of disease (ICD)-diagnoses: F00-F99 (mental and behavioral disorders) or Z56 (problems related to employment) or Z73 (problems related to life-management difficulties) at a visit to a physician or a behavioral health consultant or are prescribed any psychotropic drug (Anatomical Therapeutic Chemical (ATC) codes: N05A-C, N06A) at a participating center. These patients will be identified through their medical records. In addition, a purposive sample of patients (approximately 20) at the intervention centers will be invited to participate in interviews regarding their experience and satisfaction with care. Sampling will aim for maximum likelihood variation based on, e.g., age, sex, and education.

#### Staff recruitment

Purposive samples of health care professionals working at PCBH centers will be invited to participate in a series of interviews, e.g., about their experience working with PCBH. Sampling will aim for maximum likelihood variation based on, e.g., the center and years in practice. We estimate that approximately 15–20 interviews for each study will be sufficient to capture the study aims, as all informants will have experience with PCBH and its implementation.

#### Data collection and analysis

Various forms of data will be collected to study the three levels, patients, staff and organizations: (1) local and national registry data; (2) questionnaire data; (3) interview data; and (4) document data. Data will be stored in a pass-word protected server. All records that contain names or other personal identifiers will be stored on a similar pass-word protected server, separately from study records identified by code number. All the data analyses will be performed on pseudonymized data. The principal investigator for the study will have access to all data. Other mandated researchers within the research group will have access to pseudonymized data. A brief summary and discussion of each research question follow; see also Table 1 for the listed data sources.

##### RQ1: Effects of PCBH on patient outcomes

 National registry data on sickness absence and local registry data concerning referrals to secondary psychiatric care will be used to assess the effects of PCBH compared to standard care. Mental health problems are well documented as an important cause of sick leave, underscoring the relevance of using sick leave as a hard endpoint. Referral to secondary psychiatric care is relevant for investigating whether early PCBH-based interventions for mental health problems lower the risk of developing more severe problems due to psychiatric disorders. The effects of PCBH in terms of changes in sick leave and referral to secondary psychiatric care will be measured in the year prior to and the years after PCBH implementation. Rates for referral will be calculated using the number of visits due to mental health problems in the primary care setting and the number of patients referred to additional services in secondary psychiatric care. Data pertaining to sick leave will be obtained from registries from the Swedish Social Insurance Agency, concerning patient sick leave due to mental health problems (i.e., sick leave due to ICD10: F00-F99, Z56, Z73F). For the number of referrals, local registry data in the form of electronic medical records will be used.

##### RQ2: Characteristics of patients at PCBH centers compared to patients in standard care

Differences in sex, age, diagnostic pattern, comorbidities and prescribed medication will be investigated. Local registry data (age, sex), patient diagnoses F00-F99, Z56, and Z73F (ICD10, including specific diagnoses and combinations of diagnoses and comorbidities calculated using the Charlson Comorbidity Index) will be used. National registry data from the Swedish Prescribed Drug Registry (managed by the National Board of Health and Welfare) will be obtained concerning medication with psychotropic drugs coded as N05A, N05B, N05C and N06A according to the ATC classification system.

##### RQ3: Patient experiences with PCBH

Individual interviews (*n* = approximately 20) will capture aspects concerning patient satisfaction with the PCBH-based care, including the relationship with the behavior health consultant [32, 33]. A semistructured interview guide will be used. The interviews will be conducted on a continuous basis.

##### RQ4: Experience working with PCBH in terms of barriers to and facilitators of implementation

Individual interviews (*n* = approximately 20) with a mixture of health care professionals (physicians, nurses, and health therapists) will capture their experiences working with PCBH. A semi structured interview guide will be used, including questions on conceptualizations of PCBH and hinders at the centers for working with PCBH.

##### RQ5: Mental health therapists’ experience of their new PCBH-specific role as behavioral health consultants

Individual interviews (*n* = approximately 20) with mental health therapists (psychologists/psychotherapists) will aim to capture their experiences adopting their PCBH-specific role. A semi structured interview guide will be used. All interviews (RQ3-5) will be audio-recorded and transcribed verbatim.

##### RQ6: Work environment

Questionnaires will be administered to the staff at all PCBH centers regarding: sociodemography (age, gender, profession, year in that profession, how long the individual has worked at the primary care center), staff psychosocial factors (stress and well-being of employees measured by the Copenhagen Psychosocial Questionnaire (COPSOQ) III questionnaire), staff work commitment (measured by the Utrecht Work Engagement Scale), and exhaustion among coworkers (measured by the Karolinska exhaustion disorder scale [KEDS]). Registry data will also be used to assess sick leave among primary care staff.

##### RQ7: Accessibility of mental health care

Local registry data and document data from each included center will be used in terms of patient access to and utilization of healthcare, which are commonly reported outcomes in behavioral health research, typically with a wide variety of outcomes [33]. Several types of data will be collected: Organizational characteristics, including the number of employees, number of behavioral health consultants and physicians, number of listed patients, characteristics of the catchment area, CNI and ACG; number of visits and number of patients to behavioral health consultants; waiting times to first visit and revisits to behavioral health consultants and physicians; types of mental health interventions (e.g., focused acceptance and commitment therapy, motivational interviewing, concise CBT, behavioral activation); format of delivery (e.g., internet-based or self-help materials); and number of visits and number of patients to physicians for patients diagnosed with F00-F99, Z56, Z73F, or prescriptions of medication with ATC-codes N05A, N05B, N05C and N06A (also addresses RQ2).

##### RQ8: Cost-effectiveness of PCBH

Local registry data concerning sick leave, care accessibility, prescriptions, and health outcomes (also addressed in RQ1, RQ2 and RQ7), as well as the total cost of the implementation, collected by written documentation concerning the implementation activities undertaken during the implementation period for each of the included 24 primary care centers, will be used in the analysis of the cost-effectiveness of implementing and working according to the PCBH model. As both the costs and health outcomes of introducing the PCBH model are likely to extend beyond the 24-month study period, two analyses will be undertaken—a within-trial analysis based on data collected during follow-up as well as a long-term state-of-the-art decision-analytic model estimating expected long-term costs and health outcomes.

#### Statistical considerations

Quantitative data will be analyzed in both multilevel models and on separate levels: organization, staff and patients. Data will be collected from all centers who have initiated use of the PCBH model and in the initial analyses, all primary care centers will be considered intervention centers; i.e., analyses will be performed according to the intention to treat (ITT) principle. In addition, since features of the implementation might differ between centers, analyses will also be conducted based on centers with the highest fidelity to PCBH core components. Organization characteristics of the included primary care centers will also be collected and accounted for in the analyses, i.e., number of employees, number of behavioral health consultants and physicians, number of listed patients, characteristics of the catchment area, CNI and ACG. Furthermore, the PCBH model can be expected to require local adjustments for each primary care center. These adjustments will be carefully monitored for each participating center, and their impact on outcomes will be analyzed. In addition, data about mental health interventions (e.g., CBT) and the format of delivery (e.g., internet-based or self-help materials) will also be documented and addressed in the analyses.

#### Sample size

Approximately 3% of all patients in primary care receive an intervention by a behavioral health consultant [6, 13]. We hypothesize that this number will be at least 50% higher in the intervention group compared to the control group. A sample size based on a 50% increase, a power of 0.8 and a significance level of 0.05 yields a minimum of 1983 participants per group. Since we are using a design where heterogeneity of the populations and primary care centers probably will reduce the likelihood to detect meaningful changes, it is reasonable to double the number of participants to 3966 individuals per group. One of the pilot primary care centers has 14,841 listed patients. A study regarding Swedish primary care showed that approximately 66% of the population receives care annually at a primary care center [5]. It can thus be estimated that 9,795 (14,841 × 0.66) individual patients receive care annually at this center. If we assume that 3% [6, 13] of these patients receive a behavioral health consultant intervention, approximately 294 (9,795 × 0.03) patients annually receive a PCBH-based intervention. After the implementation of the PCBH delivery model, we estimate that this number will increase to 441 individual patients per year for one single primary care center (i.e., a 50% increase in the intervention group: 294 × 1,5 = 441). Since the project will involve 24 primary care centers, the requested sample size of at least 3966 individuals will not pose any difficulties, even considering that the number of listed patients among these centers ranges from approximately 4,500 to 21,500 listed patients (in December 2022). It should be noted that it is difficult to estimate the power of cluster studies because there can be heterogeneity within and between clusters.

#### Analysis of quantitative data

Quantitative data will be analyzed via both multilevel models and at separate levels: primary care organization, staff and patients. In the initial analyses, all primary care centers that have implemented PCBH will be considered intervention centers; i.e., analyses will be performed according to the ITT principle. Since features of the implementation might differ between centers, per-protocol analyses will also be conducted based on centers with high fidelity to PCBH core components. Differences between sexes will be investigated.

#### Analysis of qualitative data

Regarding the qualitative interview data (from patients and staff members), inductive content analysis according to Elo and Kyngäs will be employed [34]. Data analysis will include recommended steps, namely, open coding, coding sheets, grouping, categorization and abstraction. For all analyses, at least two researchers will be involved in the work to enable triangulation. In addition, NVivo or similar software will be used for data administration, sharing (between researchers) and analysis. Sex and gender considerations will be taken in account where relevant.

#### Ethical considerations

The study has been approved by the Swedish Ethical Review Authority (number 2020–05572), will be performed in accordance with the declaration of Helsinki, and is registered at clinicaltrials.gov (number NCT05633940). Health-related patient data can be considered sensitive data that warrant application to the ethical committee. Principal investigator HIL is responsible for reporting important protocol modifications in clinicaltrials.gov and other relevant parties.

For questionnaire and interview data, eligible participants will be provided with full written information about the study and the possibility of asking questions. Participants will be informed that participation is voluntary and that they can leave the study at any time point without providing an explanation, regardless of prior consent.

For registry data (medical records), informed consent will not be obtained. To increase the generalizability of the findings, registry data will be retrieved from all patients who have either been diagnosed with mental health problems or have received treatment for mental health problems at any of the participating primary care centers. Although the registry data will include sensitive data (e.g., diagnosis code, medication, comorbidity, sex and age), the Helsinki declaration states that there are situations when informed consent is not advised due to an unreasonable workload for participants. However, informed consent will be obtained from the medically responsible physician and manager at each participating center regarding the retrieval of registry data.

No human experiments will be conducted in this project. We estimate that there are minimal risks for participants (patients and staff) taking part in the project. Completing questionnaires regarding one’s health can be perceived as intrusive, and taking part in interviews can be perceived as uncomfortable. However, we believe that the benefits of this research outweigh the risks associated with participation. Indeed, mental health problems are a public health concern affecting a large proportion of the population both in Sweden and globally. This study has the potential to generate knowledge that is clinically relevant for primary care, benefiting a large proportion of the population suffering from mental health problems, thus contributing to the general good for the population and possibly providing better healthcare from a long-term perspective for the included patients. Regarding the included primary care staff, it is possible that psychosocial work environment problems will be identified, which offers the opportunity to improve the work environment for medical staff at both the intervention and control centers.

## Pilot study

The first 4 pilot centers that implemented PCHB have undergone some preliminary analyses to investigate feasibility of the project. The preliminary results are promising; in 3 of the 4 centers, the waiting lists to receive mental health care disappeared, and waiting times for visits for mental health problems decreased from 24.2 to 4.7 days (first visit) and from 22.5 to 6 days (revisits). At all the pilot centers, the number of patients who received care for mental health problems increased (a mean increase of 78%, range: 11–194%). The majority of the primary care staff at the pilot centers believed patients’ mental health care had improved and that their work had been facilitated by the implementation of PCBH, and 103 patients at the pilot centers reported being satisfied or very satisfied with the received care in a questionnaire on patient satisfaction.

## Discussion

This research project seeks to generate new knowledge about a real-world large-scale implementation and use of the PCBH model in routine primary care in Sweden. The project employs a multifaceted evaluation approach to enable an investigation of many different aspects of the PCBH model at the patient, staff and organization levels.

The project utilizes a Stepped-Wedge design, which is increasingly used in the evaluation of service delivery models [30] and was considered appropriate due to practical, logistical and resource constraints, which make it difficult to deliver the model en bloc. Stepped-wedge trials are considered more powerful than traditional parallel studies when clusters are relatively heterogeneous and/or large [31], which will likely be the case in this project. A pilot study was undertaken to determine whether the data collected to address the research questions would work as planned, demonstrating the feasibility of the research project.

An important ambition of the project is to address key knowledge gaps concerning PCBH. The planned studies have the potential to make numerous contributions to the research and practice concerning PCBH and primary care work for individuals with mental health problems. This is the first multicenter clinical trial with appropriate comparison groups. While there have been some small-scale studies of PCBH [7], there are only two published clinical trials [25, 26] and one study protocol of a pragmatic clinical trial [27] regarding PCBH. The study design will make it possible to assess many important results of the model at different levels.

The project emphasizes external validity and effectiveness rather than internal validity and efficacy. Hence, the focus is on real-world effects instead of effects under ideal, controlled conditions, which means that the project can provide a basis for decisions regarding further implementation and use of the model (or not) in other county councils. The project has the potential to generate clinically meaningful results that could be used for the future development of mental health care provision in primary care. Results will be published in international, peer-reviewed journals. Authorship will be based on the ICMJE criteria.

The research project is based on an interdisciplinary approach to stimulate an in-depth understanding of the issues under study. Mental health issues have great societal relevance but can end up “between chairs” due to their complexity and because they require knowledge from different areas or disciplines. The interdisciplinary group behind the research project consists of both researchers and clinicians, and they represent social science, medical and nursing perspectives, with professional backgrounds that include psychology and psychiatry, medicine, nursing and behavioral economics. Research shows that heterogeneous groups promote creativity since they can facilitate flexible idea generation, awareness of underlying connections between different ideas and access to more unconventional knowledge [35].

Overall, the research project will generate results that can provide a basis for decisions concerning further implementation and use of the PCBH model and has the potential to generate clinically meaningful results for the future development of mental health care provision in primary care. The project’s study design will make it possible to assess many important effects of the PCBH service delivery model at different levels (patient, staff and organization) and provide evidence of the effectiveness (or not) of the PCBH model under routine conditions in primary care.

## Data Availability

No datasets were generated or analysed during the current study.

## Abbreviations

ACG: Adjusted Clinical Groups
ATC: Anatomical Therapeutic Chemical
BHC: Behavioral Health Consultant
CBT: Cognitive Behavioral Therapy
CNI: Care Need Index
COPSOQ: Copenhagen Psychosocial Questionnaire
ICD: International Classification of Disease
ITT: Intention to Treat
KEDS: Karolinska exhaustion disorder scale
PCBH: Primary Care Behavioral Health
RQ: Research Questions
RÖ: Region Östergötland
